# Sensitive Detection of DJ-1 in Artificial Cerebrospinal Fluid Using a Portable GPTMS-Coordinated Gold Nanoparticle-Based Biosensor

**DOI:** 10.3390/bios16030146

**Published:** 2026-03-03

**Authors:** Münteha Nur Sonuç Karaboğa

**Affiliations:** Faculty of Health Sciences, Tekirdag Namik Kemal University, Tekirdag 59030, Türkiye; mnsonuc@nku.edu.tr

**Keywords:** Parkinson’s disease, DJ-1, portable biosensor, gold nanoparticles, glycidoxypropyltrimethoxysilane

## Abstract

A highly selective and sensitive compact immunosensing strategy was developed for the determination of DJ-1, a potential biomarker of Parkinson’s disease, one of the leading neurodegenerative disorders, using a portable potentiostat. Initially, screen-printed carbon electrodes (SPCEs) were modified with gold nanoparticles (AuNPs), followed by functionalization with 4-mercapto-1-butanol (MOH). Subsequently, the AuNPs-doped and hydroxyl-functionalized electrodes were treated with 3-glycidoxypropyltrimethoxysilane (GPTMS) to facilitate immobilization of anti-DJ-1 antibodies. Immobilization steps were monitored using electrochemical impedance spectroscopy (EIS) and cyclic voltammetry (CV) performed on a bench potentiostat, while the entire analytical performance of the developed biosensor system and its response in artificial cerebrospinal fluid (aCSF) were evaluated by monitoring cathodic current changes with a portable electrochemical reader. The resulting biorecognition element enabled the detection of DJ-1 within the concentration range of 0.001 to 0.3 ng/mL, based on cathodic current changes, achieving a limit of detection as low as 0.00059 ng/mL. Surface morphology and elemental composition alterations were characterized by scanning electron microscopy (SEM), Fourier transform infrared spectroscopy (FTIR), and energy-dispersive X-ray spectroscopy (EDX). A notable advantage of this GPTMS@AuNPs-based biosensor system is its prolonged storage stability and its capability to accurately quantify DJ-1 in artificial cerebrospinal fluid samples, with recovery rates ranging from 98.66% to 123.3%.

## 1. Introduction

Neurodegeneration, defined as the selective, progressive, and irreversible loss of neurons, subsequently leads to neuronal disorders [1]. Parkinson’s disease (PD), a neurodegenerative disorder associated with loss of dopaminergic neurons and Lewy bodies, is accompanied by motor and behavioral disorders such as resting tremor, postural instability, rigidity, and slowing of voluntary movement [2,3,4]. Unfortunately, PD is not recognized in the early stages in most cases [5]. By the time obvious symptoms appear, approximately 80% of dopaminergic neurons have been lost [6] Therefore, understanding this disease in the early stages will contribute positively to the course of the disease and the treatment process. Easily accessible, affordable, and accurate diagnostic biomarkers have the potential for widespread clinical use, including by primary caregivers, and allow for the optimization of treatment strategies, provision of appropriate support and care, and reduction of healthcare costs [7,8,9].

At this point, different studies have been conducted, examining different proteins—and, therefore, biomarkers—related to the disease, providing very valuable information in terms of the mechanism and elucidation of PD. Among these findings, the DJ-1 protein encoded by the *PARK7* gene is quite remarkable. DJ-1, a 19.8 kDa dimeric form reflecting a helix–strand sandwich model [10], is a protein that intervenes in transcription and translation processes at a multi-layered level and plays a role in cellular processes [11], which includes protecting cells against oxidative stress-induced cell death [12]. The pleiotropic roles of DJ-1 partly explain why DJ-1 dysregulation leads to such widespread pathological consequences [13]. Autopsy studies have shown that DJ-1 also forms high-molecular-weight complexes predominantly in brain regions, including the substantia nigra [14].

Increased CSF and plasma concentrations of DJ-1 have been reported in the brains of PD patients surrounding Lewy bodies [15,16,17,18,19]. Moreover, studies on post-mortem brain tissue [20,21] and other biological fluids [22,23] have shown increased expression of the oxidized form of DJ-1 compared to healthy controls.

All these studies are sprouting other new studies and aim to understand the role of DJ-1 in PD pathophysiology in more detail from a biomarker perspective. The available data emphasize that DJ-1 is a potential biomarker for PD. This study focused on the determination of the DJ-1 protein using disposable electrodes and a portable potentiostat, based on the synergy between nanoparticles and self-assembled monolayers derived from silane chemistry.

In addition to the many known advantages of electrochemical systems in analyzing analytes from a wide perspective, such as the quality of water, food, and pharmaceutical compounds, it can provide many remarkable points, such as the integration of portable feature, without requiring a special laboratory environment for monitoring analytes, supporting personnel medicine, and easing the economic burden of states in health. A number of detection methods, including electrochemical impedance spectroscopy (EIS), voltammetry, and amperometry, can be used with portable potentiostats [24] Based on the affinity binding between antibodies and antigens, nanoparticle-based electrochemical immunosensors are attractive and promising screening tools that enable the precise detection of a wide range of targets, including proteins, hormones, and antibodies [25]. In order to successfully immobilize the bioreceptor or target molecules, the use of nanoparticles in the construction of such devices significantly increases the transducer surface area, sensitivity, and selectivity. The design and production of nanoparticles in recent years has mostly concentrated on utilizing the synergistic effects that result from the coupling of several materials [26]. Since AuNPs are easily conjugated with many biomolecules, they are great candidates for use as labels in the electrochemical detection of various proteins. AuNPs have also been discovered to exhibit remarkable electrocatalytic activity toward other chemical reactions [27]. This work attempted to enhance the efficacy of gold nanoparticles in producing biorecognition elements by employing 3-glycidoxypropyltrimethoxysilane. Biomolecules can be immobilized by first activating their surfaces with a silane reagent, which creates new reactive sites for attachments later on. A silanol-covered surface can form a covalent bond thanks to the silane. 3-glycidoxypropyltrimethoxysilane is frequently used over alternative coupling agents due to its increased immobilization density of oligonucleotide probes and repeatability [28,29].

In this study, we developed a portable electrochemical immunosensor on screen-printed carbon electrodes, functionalized with gold nanoparticles and silane linkers, for sensitive and selective detection of DJ-1, a potential biomarker of Parkinson’s disease. The sensor demonstrated excellent analytical performance, including a low detection limit, a wide linear range, high selectivity, and reproducibility. Surface modifications and antibody immobilization were thoroughly characterized using SEM, EDX, and FTIR, confirming effective sensor construction. Practical applicability was validated in artificial cerebrospinal fluid, highlighting the potential of this platform for clinical diagnostics.

## 2. Materials and Methods

### 2.1. Reagents and Instruments

Gold (III) chloride trihydate 4-mercapto 1-butanol, glycidoxypropyltrimethoxysilane, N-(3-dimethylaminopropyl)-N′-ethylcarbodiimide (EDC), N-hydroxysuccinimide (NHS), and the other reagents were supplied from Sigma-Aldrich (St. Louis, MO, USA). Monoclonal Anti-DJ-1 antibody produced from rabbit, DJ-1 obtained from human plasma, and bovine serum albumin (BSA) were supplied by Sigma-Aldrich, St. Louis, MO, USA. Artificial CSF obtained from R&D Systems (Minneapolis, MN, USA). DJ-1, 1% bovine serum albumin (BSA), and anti-DJ-1 solutions were prepared using a phosphate buffer system (50 mM, pH 7.0) and stored at −20 °C until utilized. As a redox probe solution, phosphate buffer containing 1 M KCl, 5 mM [Fe(CN)_6_]^4−^, and 5 mM [Fe(CN)_6_]^3−^ was used in all electrochemical studies.

The formation and disruption of chemical bonds during each step of biosensor development were monitored using FTIR spectral analysis in the range of 4000–400 cm^−1^. Immobilization steps were monitored by SEM (Axia Lovac Chemisem, Thermo Scientific, Waltham, MA, USA).

Screen-printed carbon electrodes (SPCE/110) were purchased from DropSens (Metrohm, Spain) and consist of carbon working and auxiliary electrodes, with a silver reference electrode (the basic dimensions of the strip are 3.4 × 1.0 × 0.05 cm).

The design and immobilization steps of the biosensor were followed using the commercial Gamry Potentiostat/Galvanostat, Interface 1000 system (Gamry Instruments, Warminster, PA, USA), including CV and EIS software version 7.0.

For our electrochemical investigations, we purchased a portable, potentiostat-based Dropstat Electrochemical Reader from Metrohm AG, Herisau, Switzerland. This device can show the analyte concentration on an LCD screen that is customized based on variations in cathodic current.

### 2.2. Functionalization of Gold Nanoparticles and Self-Assembly of Thiols

Before starting the study, bare SPCEs were cleaned three times with ethanol and double distilled water and dried with pure argon gas. AuNPs were obtained using sodium borohydride as reported in our previous studies [30] briefly a 500 mL colloidal gold suspension (0.2 mM) was prepared by diluting 1 mL of 0.1 M HAuCl_4_ in distilled water. While stirring, 10 mL of 0.13 M NaBH_4_ was slowly added dropwise to reduce the gold ions. During the reduction, the solution exhibited a characteristic color shift from yellow to ruby red, indicating successful nanoparticle formation. The average diameter was found to be 40 nm by SEM.

KOH (1% *w*/*v* in pH 7.0, 50 mM PBS) was added dropwise to the clean electrode surfaces to generate active –OH groups on the bare SPCEs surface. The electrodes were then allowed to air-dry for 30 min at room temperature (SPCE/OH) [31]. In the next step, AuNPs solution was added dropwise to the electrodes containing –OH groups and incubated at room temperature for 60 min (SPCE/OH/AuNPs). After washing and gentle drying, SPCEs doped with AuNPs were incubated with 5% (*v*/*v*) mercaptobutanol in ethanol for 60 min to form self-assembled monolayers containing –OH terminals. After incubation, the SPCEs a self-assembled monolayer (SAMs) were cleaned with ethanol, followed by pure water, and then gently dried (SPCE/OH/AuNPs/MOH).

### 2.3. Covalent Attachment of Anti-DJ1 to AuNPs-Doped@MOH@GPTMS

**Figure 1:**
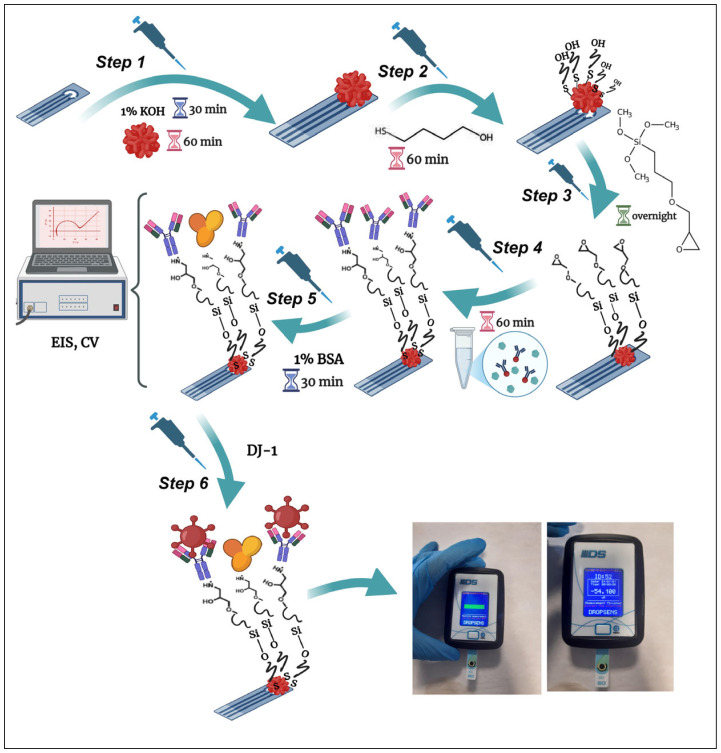
Stepwise schematic illustration of the fabrication and application of the DJ-1 biosensor. Created with BioRender.com.

A 1% (*v*/*v*) GPTMS solution in toluene was applied to the surface of SPCEs pre-modified with AuNPs and terminal –OH groups (derived from MBOH), and incubated overnight at room temperature to allow for the formation of an epoxy-silane –self-assembled monolayer bearing terminal epoxy groups capable of reacting with the amine groups of anti-DJ-1. Following incubation, the resulting SAMs-modified electrodes (SPCE/OH/AuNPs/MBOH/GPTMS) were sequentially washed with toluene and double-distilled water to remove unbound GPTMS molecules and subsequently dried under a stream of pure argon. The –COOH groups of the anti-DJ-1 antibody were activated using the heterobifunctional cross-linker reagent EDC (as a coupling agent) and -NHS (as an activator). Anti-DJ-1 solution containing 0.4 M EDC and 0.1 M NHS was prepared in a 2:1:1 ratio and stored at 4 °C for 60 min for activation. At the end of the period, 20 µL of the activated antibody solution was added dropwise to the AuNPs doped@GPTMS SPCEs surfaces and incubated at room temperature for 1 h. Routine washing and drying procedures were performed after incubation. Finally, 1% (*w*/*v*) BSA was added dropwise to the SPCEs surfaces to block the nonspecific binding sites of the generated biorecognition element and incubated at room temperature for 30 min. After incubation, the SPCEs were rinsed with double-distilled water and dried with ultrapure argon gas and were ready for DJ-1 determination. Figure 1 schematizes the immunosensor’s fabrication procedures.

### 2.4. Electrochemical Evaluation of Immunosensor and DJ-1 Detection

The immobilization process of the developed immunosensor was systematically characterized using parameters established in our previous studies via cyclic voltammetry (CV; potential range: −500 mV to +1000 mV, scan rate: 100 mV s^−1^, step size: 10 mV) and electrochemical impedance spectroscopy (EIS; initial frequency: 50 kHz, final frequency: 0.05 Hz, number of points: 5, formal potential: 0 V). DJ-1 detection was conducted by applying varying concentrations of DJ-1 solutions to the AuNPs@GPTMS-modified SPCE surface, followed by incubation for 60 min at room temperature. Subsequently, the electrode surface was rinsed with PBS, a redox probe was introduced, and the cathodic current response was recorded using a Dropstat portable potentiostat. The resulting peak current (Ipc) values were employed to construct the calibration curve of the immunosensor, enabling quantitative evaluation of DJ-1.

### 2.5. Monitoring of DJ-1 in Artificial CSF

The practical application of the immunosensor was tested by using artificial cerebrospinal fluid samples (R&D Systems, 10 times diluted with pH 7.0 PBS). To evaluate the quantitative performance of the GPTMS/MOH/AuNPs@SPCE biosensor in an artificial cerebrospinal fluid (aCSF) matrix, the standard addition method combined with spike recovery experiments was applied.

In the standard addition protocol, predetermined concentrations of the target analyte, DJ-1, were spiked into a protein-free aCSF solution, ensuring a constant total sample volume across all conditions. Electrochemical measurements were carried out using the DropStat electrochemical reader, and the cathodic current response of the biosensor was recorded under optimized conditions. The resulting current–concentration data were analyzed via linear regression. Method precision was assessed by calculating the relative standard deviation (RSD%), and analytical accuracy was evaluated through recovery analysis, based on the ratio of calculated to spiked concentrations.

## 3. Results

### 3.1. Electrochemical Confirmation of Immobilization Procedures

**Figure 2:**
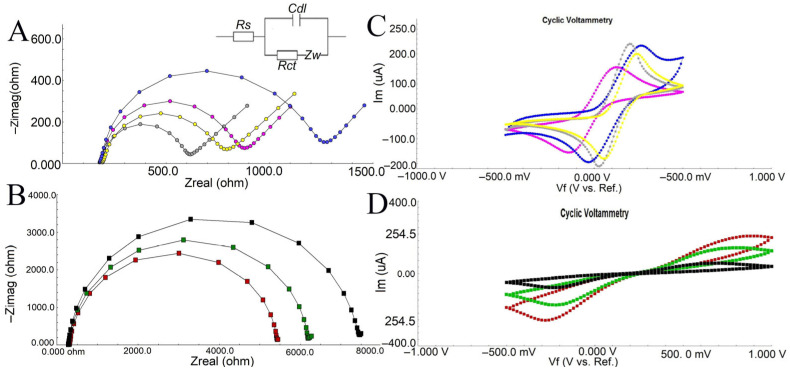
Electrochemical impedance spectra (**A**,**B**) and cyclic voltammograms (**C**,**D**) for immobilization steps of anti-DJ1. [grey: bare SPCEs, yellow: SPCE/OH/AuNPs, pink: SPCE/OH, blue: SPCE/OH/AuNPs/MOH, red: SPCE/OH/AuNPs/MOH/GPTMS/anti-DJ1, green: SPCE/OH/AuNPs/MOH/GPTMS/anti-DJ1/BSA, black: SPCE/OH/AuNPs/MOH/GPTMS]. Equivalent circuit model inserted in (**A**).

Electrochemical changes occurring during the immobilization steps of the immunosensor were followed by EIS and CV. The electron transfer resistance is triggered by chemical changes on the electrode surface during the development of biorecognition elements. EIS can characterize these changes without the need for labeling. In order to improve the reactivity of subsequent processes on the SPCE strips’ surface, active –OH groups were initially generated. To expand the surface area, AuNPs were then adsorbed on the surface. Due to their large and specific surface area and easy conjugation with biomolecules through the Au-S bond, AuNPs are often used as immobilization platforms to bind a large number of biomolecules, as in this study. SPCEs modified with AuNPs were incubated with 4-mercapto 1-butanol to ensure both Au-S conjugation and to form a new active –OH group at the terminal. The packed state of the self-assembled monolayer formed with 4-MOH is an important step for the sensitivity of the biosensing unit (Figure 2).

The electrodes’ charge transfer resistance (Rct), which changes according to the characteristics of the electrode surface, is indicated by the semicircle of the Nyquist plot. The EIS diagram in Figure 2A indicates that bare SPCE exhibits highly conductive properties. When SPCE surfaces are activated with

–OH, conductivity is still preserved, but electron transfer to the surface is partially impaired due to the repulsion of the [Fe(CN)_6_]^3−^/4-redox probe by the negative hydroxyl groups. After the adsorption of AuNPs onto the surface, the charge transfer resistance decreases significantly due to the increased conductivity. Another important element of EIS, the Warburg impedance (Zw), represents the delay caused by the diffusion of electroactive species onto the electrode. The diffusion of reactants onto the electrode surface is a slow process that can only occur at low frequencies. At high frequencies, the reactants do not have enough time to diffuse. The 45-degree slope observed after the modification with AuNPs may indicate that this step is diffusion controlled. Gold nanoparticles allow immobilization of larger amounts of protein compared to non-nano gold while also increasing sensitivity. In addition, thiol-modified gold nanoparticles can be used as biosensing platforms by developing a hybrid nanomaterial that possesses both the highly selective recognition properties of bimolecular antibodies and the unique electronic and photonic properties of nanoparticles. It is expected that strong Au-S conjugation will occur as a result of the modification of AuNPs-modified SPCEs with 4-MOH. Treatment of SPCEs with 4-MOH provides a tightly packed SAMs structure and prepares the ground for the next step with the terminal –OH group. This situation is reflected in the Nyquist plot as an increase in charge transfer resistance due to both the regular structure of SAMs and the repulsion of the redox probe by the terminal –OH groups. After the formation of active –OH groups with 4-MOH on the surface of SPCEs modified with AuNPs, SPCEs were incubated with GPTMS overnight. Each –OH group can react with Si atoms and form a dense monolayer that does not allow cross-linking. In order to prevent cross-linking of silane groups, the maximum number of hydroxyl groups should be formed. The non-conductive nature of the GPTMS layer causes the epoxy silane monolayer to act as a barrier, preventing the diffusion of the redox mediator onto the electrode surface. This phenomenon is evidenced by a significant enlargement of the semicircle in the Nyquist plot, indicating increased impedance (Figure 2B). Following the immobilization of anti-DJ-1, a decrease in charge transfer resistance is observed. This reduction may be attributed to the shorter electron tunneling distance between the electrode surface and the anti-DJ-1 molecules, which act as mediators that facilitate electron transfer to the surface. The interaction between the cationic regions of the anti-DJ-1 antibody and the negatively charged redox probe promotes electron transfer along the electrolyte–electrode interface. In the final step, BSA blocking—applied to prevent nonspecific binding and to passivate remaining active sites—led to the formation of an additional barrier on the surface. The binding of BSA increased both the capacitance and resistance of the system, resulting in higher impedance values, which confirms the successful fabrication of the anti-DJ-1/GPTMS/MOH/AuNPs/SPCE biosensor (Figure 2B). EIS results are summarized in Table 1. The bare SPCE exhibited a charge transfer resistance (Rct) of 481.4 ± 9.49 Ω, which slightly increased after hydroxyl functionalization (SPCE/OH, 600.5 ± 6.5 Ω), confirming the presence of the OH layer. Upon modification with AuNPs, Rct decreased to 492.5 ± 8.1 Ω, indicating improved electron transfer due to the high conductivity and large surface area of AuNPs. Subsequent modification with MOH led to a significant increase in Rct (1350 ± 25.59 Ω) along with a measurable double-layer capacitance (Cdl = 1.00 ± 0.001 µF), suggesting successful surface coverage. After introducing GPTMS, Rct drastically increased to 7100 ± 86.42 Ω, while Cdl slightly increased to 1.26 ± 0.0013 µF, demonstrating the formation of a silane-based layer and decreased electrode conductivity. Immobilization of anti-DJ1 antibodies reduced Rct to 4520 ± 63.99 Ω but caused a remarkable increase in Cdl (2.23 ± 0.0024 µF), confirming effective antibody attachment and enhanced dielectric properties. Finally, blocking with BSA resulted in a moderate increase in Rct (5470 ± 66.07 Ω) and a decrease in Cdl (1.31 ± 0.0013 µF), consistent with the successful prevention of nonspecific binding and stabilization of the biosensor surface. These results collectively confirm the stepwise fabrication process and validate the effective modification of SPCE for the development of the DJ-1 biosensor.

The peak potential separation, which is inversely proportional to the electron transfer rate, is used for the electrochemical evaluation of the electrode conductivity. According to the equation ∆Ep = (Epc − Epa), the peak potential separation between anodic and cathodic waves can be calculated. According to this equation, while the ∆Ep value of bare SPCEs functionalized with –OH was 0.27 V at a scan rate of 100 mV/s, the ∆Ep value decreased to 0.18 V after AuNPs were deposited on the surface. This decrease indicates that AuNPs greatly increased the conductivity of the electrode. When a highly insulating self-assembled monolayer was formed with GPTMS, ∆Ep increased to 1.17 V, while the ∆Ep value was 0.92 V in the anti-DJ1 immobilization step due to the electron tunneling effect. EIS and CV data are consistent with each other and support the formation of a successful biorecognition unit (Figure 2C,D).

### 3.2. SEM, EDX and FTIR Analysis

**Figure 3:**
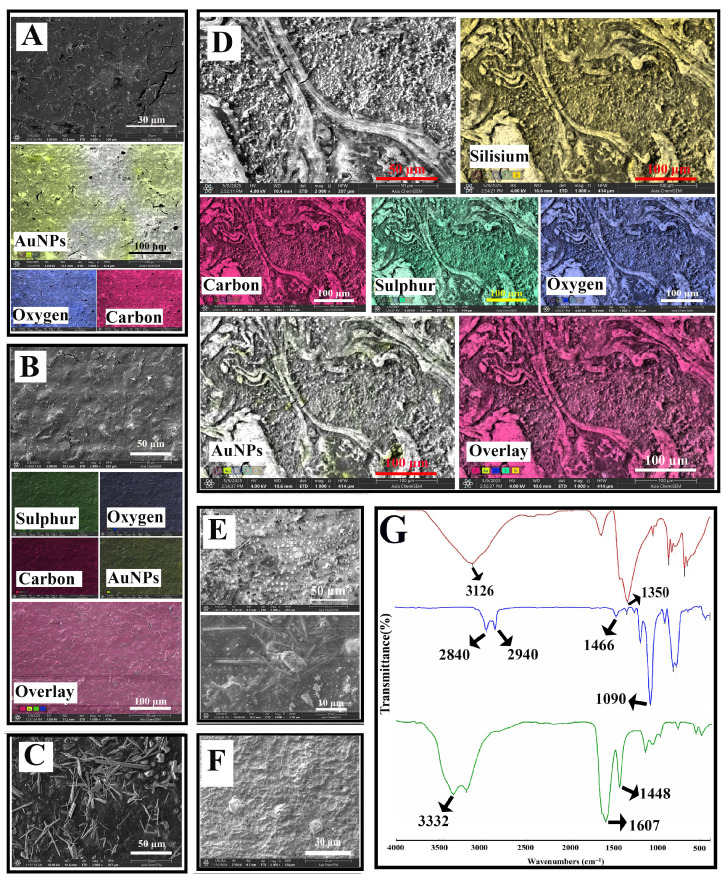
EDX and SEM analysis of AuNPs adsorption (**A**), 4-MOH modification (**B**) and GPTMS SAMs generation onto the modified surface (**D**). SEM images of GPTMS modification onto non-modified surface (**C**), anti-DJ1 immobilization ((**E**); 2000× and 8000×, respectively) and BSA blocking (**F**). FTIR spectra of MOH modification ((**G**); red), SAMs of GPTMS generation ((**G**); blue) and anti-DJ1 immobilization ((**G**); green). Scale bars are indicated in each panel.

The surface elemental characteristics of the modified SPCEs were analyzed and mapped using the SEM-EDX mapping technique to verify the homogeneous distribution of nanomaterials on the SPCEs’ surface (Figure 3). Although the SPCE/OH/GPTMS step was not directly included in the biosensor design, the SEM images of the modified electrode were also analyzed to better understand the other steps. Figure 3A presents the SEM and EDX data from the first step of the biosensor system designed for the determination of DJ-1. While gold nanoparticles appeared as spots on the surface in SEM pictures, EDX mapping indicated a greater quantity of gold nanoparticles in comparison to bare electrodes. After the AuNPs modified SPCEs were treated with 4-mercaptobutanol, it was observed that the surface was covered with a film layer. The elemental distribution of 4-MOH-modified AuNPs@SPCEs contained sulfur elements, unlike the previous step, and the gold content on the surface decreased (Figure 3B). Figure 3C presents SEM images of SPCEs initially functionalized with hydroxyl (–OH) groups and subsequently modified with GPTMS. The distinct rod-shaped structures observed on the surface are attributed to the self-assembly of GPTMS. In Figure 3D, SEM images of GPTMS-treated MOH/AuNPs@SPCEs reveal prominent fork-like features and dispersed nanoparticles on the surface. Comparative analysis of Figure 3C,D suggests that these fork-like structures result from the aggregation and alignment of GPTMS into rod-like formations. Elemental mapping of the GPTMS-modified surface indicates a significant decrease in sulfur and gold content compared to the previous modification step, while an increase in silicon (Si) signals confirms the successful incorporation of GPTMS. Following the immobilization of anti-DJ1 to form the biorecognition element, Figure 3E (at 2000X and 8000X magnification) displays typical globular antibody formations on the surface. Finally, Figure 3F illustrates the impact of BSA, used as a blocking agent, which leads to the development of a dense protein layer, altering the surface morphology once again. Figure 3G presents the FTIR spectra corresponding to the sequential surface modifications: from top to bottom, 4-MOH functionalization, GPTMS layer formation, and anti-DJ1 immobilization. In the first spectrum, the broad peak observed around 3200 cm^−1^ is attributed to the O–H stretching vibration of mercaptobutanol. The band at approximately 1350 cm^−1^ likely corresponds to C–H bending modes. Upon modification with GPTMS, the middle spectrum reveals characteristic forked peaks around 2900 cm^−1^, which can be assigned to symmetric stretching vibrations of –CH_3_ and –CH_2_ groups present in the GPTMS structure. Additionally, the bands around 1400 cm^−1^ are indicative of epoxy ring stretching vibrations, while the prominent band at 1090 cm^−1^ is attributed to Si–O–CH_3_ stretching of the methoxy groups in GPTMS. Among the peaks obtained from the immobilization of anti-DJ1, the vibration band at 3330 cm−1 is the N-H stretching band of the secondary amine. The most obvious FTIR information regarding the covalent immobilization of anti-DJ- belongs to the amide peak at 1640 cm^−1^.

### 3.3. Optimization of Fabrication Parameters for Bioelectrodes

To enhance the biosensor design’s performance, the following parameters were optimized: the effects of MOH concentration (1, 5, 10% *v*/*v*), GPTMS concentration (0.5, 1.0, 2.0% *v*/*v*), and anti-DJ1 concentration (20, 100, and 200 ng/mL) on the biosensing responses (Supplementary Materials). When 1% MOH was used, the biosensor showed no response, whereas the regression coefficient decreased significantly at 10% MOH. Therefore, 5% MOH was selected as the optimal concentration for subsequent surface modifications (Figure S1). In the case of GPTMS, using a high concentration (2%) combined with overnight incubation appeared to damage the SAM layer and promote unwanted side interactions, as reflected in the reduced biosensor response (Figure S2). Conversely, at low GPTMS concentrations, the formation of a densely packed SAM layer was likely insufficient, leading to a lower regression coefficient. Optimization studies for anti-DJ1 immobilization revealed that the biosensor exhibited the best performance when 100 ng/mL of anti-DJ1 was used, yielding a high regression coefficient (R^2^ = 0.99).

### 3.4. Analytical Performance of the GPTMS/MOH/AuNPs@SPCEs

#### 3.4.1. Linear Dynamic Range, Repeatability, and Reproducibility Utilizing Portable Potentiostat

**Figure 4:**
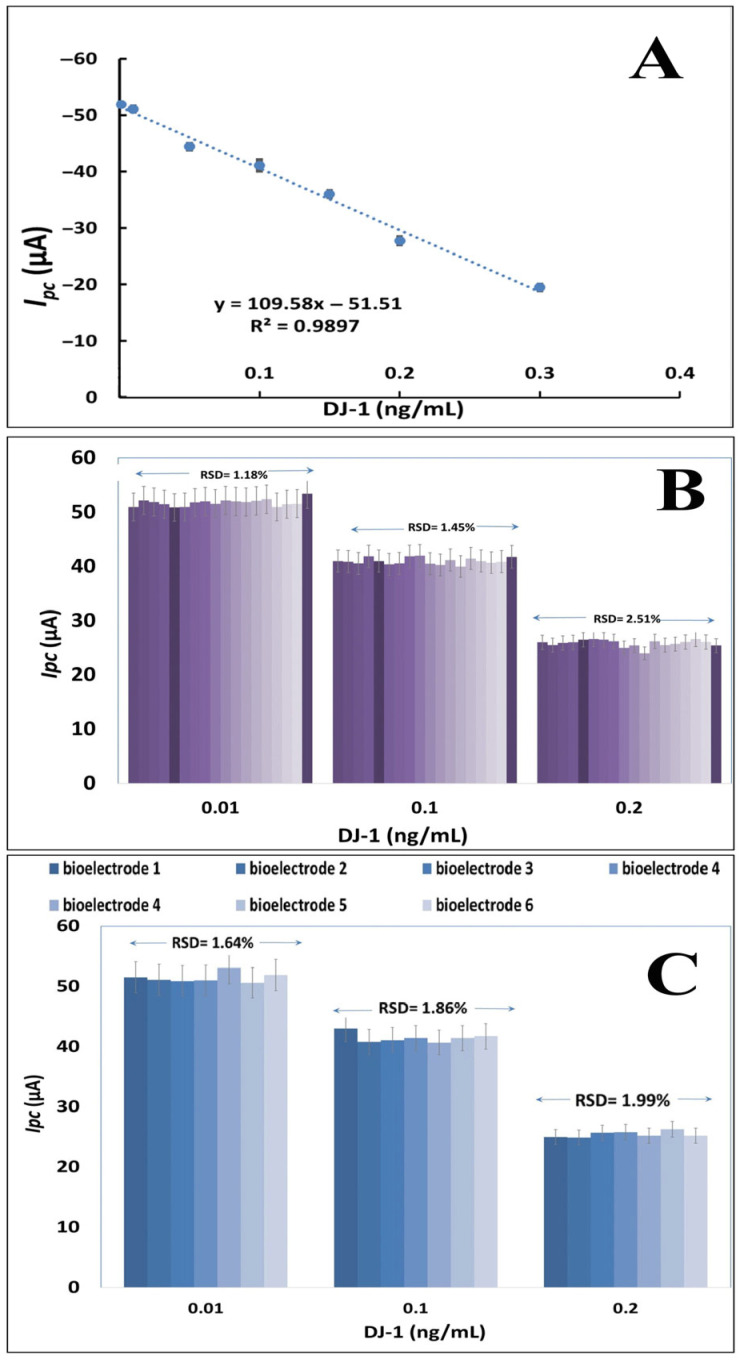
The calibration plot between the cathodic peak current and DJ-1 concentrations from 0.001–0.3 ng/mL (**A**) repeatability of the GPTMS/MOH/AuNPs@SPCEs for DJ-1 detection at the same electrode (**B**). The reproducibility of the GPTMS/MOH/AuNPs@SPCEs for DJ-1 detection at the six different preparations of electrodes (**C**).

The concentration of DJ-1 was determined by the change in cathodic current using a portable potentiostat at the GPTMS/MOH/AuNPs@SPCEs under optimum conditions. The standard calibration curves of DJ-1 were plotted against the current responses with DJ-1 concentrations in the range of 0.001–0.3 ng/mL (Figure 4A). The linear regression equation was expressed as y = 109.58x − 51.51 (y  =  current in μA and x  =  DJ-1 concentration in ng/mL), and the linear correlation coefficient was 0.9897. The limit of detection (LOD based on 3(Sa/b)) and the limit of quantification (LOQ based on 10(Sa/b)) were calculated as 0.00059 and 0.0019 ng/mL, respectively. Sa is the standard deviation of the blank sample, and b is the slope of the calibration plot. The developed immunosensor surface design provides a wide detection range. The LOD of this method (0.00059 ng/mL) is 100 times lower than the lowest LOD value in the ELISA results presented in Table 2. In addition, the biorecognition element exhibited high performance due to the presence of AuNPs and GPTMS-induced surface epoxy groups. Furthermore, biosensor systems developed for the determination of DJ-1 are quite limited. In the MIPPy-based biosensor system, the determination range is 0.02–10 ng/mL, and the LOD is 0.02 ng/mL. In another study conducted by our research group for DJ-1, nanocomposite-based ITO electrodes were used with a bench potentiostat, and the determination range for DJ-1 was quite sensitive and was 4.7 fg mL^−1^ to 4700 fg mL^−1^ [32].

In addition to the high sensitivity of the presented study, it also offers advantages such as practical preparation and the ability to determine the target molecule with a portable device. The repeatability of the developed biosensor was evaluated by comparing the cathodic current responses of GPTMS/MOH/AuNPs bioelectrodes developed under the same conditions with DJ-1 at concentrations of 0.01, 0.1, and 0.2 ng/mL. For each concentration, 6 measurement sets were generated (*n* = 3, 18 measurements were taken for each concentration). As a result of the repeatability studies, the RSD values were calculated as 1.18%, 1.45%, and 2.51%, respectively (Figure 4B). The system was also tested for reproducibility by preparing six different bioelectrodes with the same procedure.

Reproducibility can be defined as the ability of the biosensor to produce the same responses for a replicated experimental set. The RSDs of the reproducibility studies ranged from 1.64% to 1.98 (Figure 4C). RSDs being better than acceptable values show that the developed GPTMS/MOH/AuNPs@SPCE biosensing system works consistently and the random error margin is low. Expansion of the surface area with gold nanoparticles and GPTMS increased the sensitivity of the biorecognition element to form immunocomplexes with DJ-1.

#### 3.4.2. Selectivity and Storage Stability Performance

**Figure 5:**
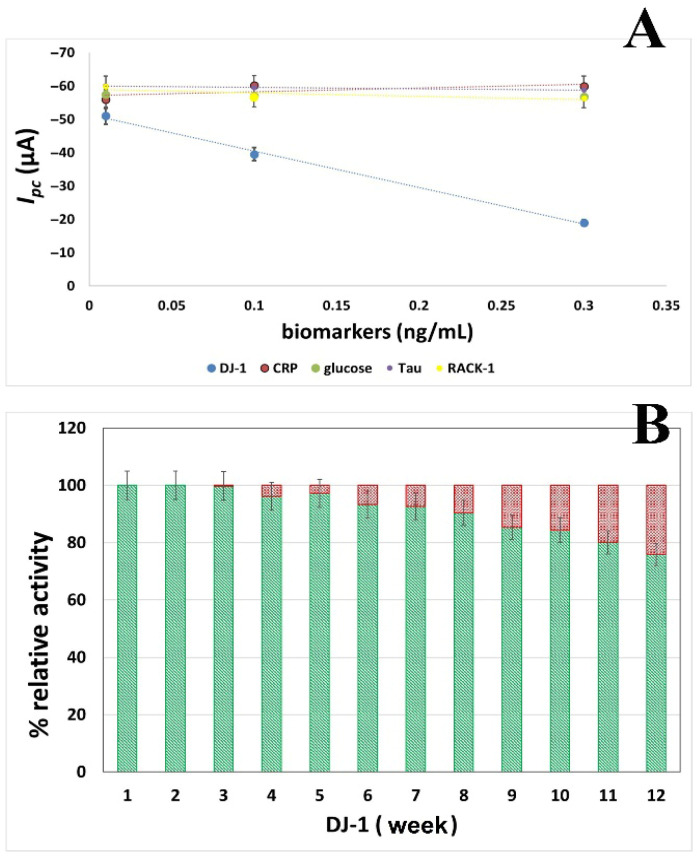
Selectivity of the developed biosensor toward different concentrations of CRP, glucose, Tau, and RACK-1 (**A**), and the storage stability of the biosensor for DJ-1 detection over 12 weeks (**B**).

A bioreceptor’s selectivity is its capacity to identify a particular analyte in a sample that contains additional additives and impurities. The selectivity of the immunosensor was analyzed in the presence of different biomarkers (CRP, glucose, Tau, RACK-1) in a phosphate buffer. For the selectivity study, the biomarkers were chosen from the set of analytes readily available in our laboratory. This selection allowed a preliminary evaluation of the sensor’s response to non-target analytes under controlled conditions. Although the selection was based on practical availability rather than clinical priority, this approach provides initial insights into selectivity, and future studies will focus on clinically relevant biomarkers to further validate sensor performance. Biomarkers were prepared at three standard concentrations (0.01, 0.1, and 0.3 ng/mL) and analyzed using a portable potentiostat with the developed BSA/anti-DJ-1/GPTMS/MOH/AuNPs@SPCE bioelectrodes. Before treatment with biomarkers, cathodic peak current blank measurements of bioelectrodes were recorded (−58.8 ± 0.00622 µA, *n* = 3). When Figure 5A is examined, while the specific interaction between anti-DJ-1 and DJ-1 is clearly reflected in the signal, the contribution of other biomarkers is quite minimal due to their inability to provide immunospecificity. The interference ratio for each interference compound ranged from 0.8% to 1.2%, with an average interference level of approximately 1%. These low interference ratios support the selectivity of the sensor/analytical method. The regression equations obtained for the interference tests in Figure 5A were as follows: Tau y = 4.0318x − 59.9844 (R^2^ = 0.9857), RACK-1 y = 10.514x − 59.004 (R^2^ = 0.5951), CRP y = −11.044x − 57.157 (R^2^ = 0.5029), and glucose y = 2.1785x − 57.398 (R^2^ = 0.8044); these results indicate that the interference effect is minimal.

Selectivity results show that the biosensor design is quite good at determining the DJ-1 target molecule; however, it creates a stopper effect on other interferences. Although the current selectivity performance is sufficient for practical applications, it can be further enhanced by employing more advanced blocking agents (e.g., casein or PEGylated compounds instead of BSA) to suppress residual nonspecific binding. In addition, oriented antibody immobilization strategies may help improve antigen–antibody recognition efficiency. These approaches will be considered in our future studies to further increase the selectivity of the proposed biosensor.

For the storage stability assessment, BSA/anti-DJ-1/GPTMS/MOH/AuNPs@SPCE bioelectrodes were stored at 4 °C and tested weekly for DJ-1 detection at a constant concentration (0.05 ng mL^−1^; *n* = 3) at the same time of day for a period of 12 weeks. The results indicated that the bioelectrode response decreased to 80.06% of its initial value after 10 weeks, demonstrating that the proposed biosensor exhibits satisfactory long-term storage stability (Figure 5B)

#### 3.4.3. Applications of the Developed Biosensor in Artificial Cerebrospinal Fluid Samples

**Figure 6:**
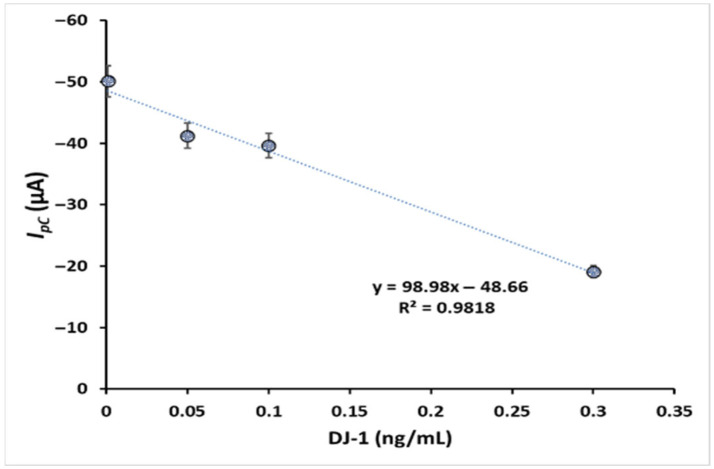
A calibration plot of DJ-1 obtained using the standard addition method.

DJ-1 analysis was performed in artificial CSF (final ion concentrations (in mM): Na^+^ 150; K^+^ 3.0; Ca^2+^ 1.4; Mg^2+^ 0.8; P 1.0; Cl^−^ 155) to test whether there is a matrix effect, and the standard curve was generated using artificial CSF (Figure 6). The results are presented in Table 3. The %RSDs obtained as a result of repeated measurements for each DJ-1 concentration are less than 10% and within acceptable values. The fact that the obtained recovery is within the acceptable range shows that the GPTMS/MOH/AuNPs@SPCEs biosensor can work accurately and reliably in complex matrices such as artificial CSF. The formation of SAMs with GPTMS provided a homogeneous layer on the defined surface. In addition, the fact that GPTMS acts as a natural cross-linker due to the Si-O it contains provides a significant contribution to the formation of biorecognition elements. In addition, the epoxy groups in the GPTMS structure are highly reactive to nucleophiles such as thiols and amines in aqueous environments and are relatively stable at neutral pH [39]. The functionalization of the SPCE surface with AuNPs and then the blending of the superior properties of GPTMS provided the analytical performances of the DJ-1 assay, such as high selectivity, sensitivity, reproducibility, and relatively long storage life. Although the number of presented datasets appears limited, the biosensor performance has been rigorously validated through multiple critical parameters. The device achieved an ultra-low detection limit of 0.00059 ng/mL (0.59 pg/mL), which is highly competitive compared to reported biosensors. Reproducibility was verified with low RSD values (<7%) across independent experiments, while recovery values ranging from 98 to 125% in spiked CSF samples confirmed the accuracy in complex biological matrices. Furthermore, long-term storage stability studies demonstrated that the biosensor retained a high percentage of its initial response over several weeks. Collectively, these results provide strong technical evidence for the biosensor applicability, supporting its practical use without the need for excessive additional datasets.

## 4. Conclusions

Early diagnosis of Parkinson’s disease, one of the most prevalent neurodegenerative disorders, is crucial for improving patients’ quality of life and for the development of novel therapeutic strategies. The proposed system, designed for the highly sensitive and selective detection of DJ-1—a potential biomarker of the disease—is compact, allows for on-site analysis, and offers long-term storage stability. The immobilization steps of the bioelectrode fabrication were optimized using a bench-top potentiostat, while its analytical performance characteristics were evaluated using a portable electrochemical reader, highlighting the system’s applicability in point-of-care settings. The developed bioelectrode, based on GPTMS/MOH/AuNPs@SPCEs, demonstrated stability within the linear dynamic range of 0.001–0.3 ng/mL, even in the presence of potential interferents, by exhibiting a consistent cathodic response in an artificial cerebrospinal fluid environment. In this context, it presents a promising alternative to conventional methods such as ELISA and holds significant potential for clinical applications. Additionally, sensor-based techniques for the detection of this key protein remain limited in the literature.

## Figures and Tables

**Table 1:** Impedance characteristics of bare and modified SPCE electrodes in the development of the DJ-1 immunosensor.

Biosensor	Rct/Ω	Ru/Ω	Cdl/µF
bare SPCE	481.4 ± 9.49	179.7 ± 2.18	–
SPCE/OH	600.5 ± 6.5	255.4 ± 2.2	–
SPCE/OH/AuNPs	492.5 ± 8.1	300.7 ± 2.4	–
SPCE/OH/AuNPs/MOH	1350 ± 25.59	507 ± 5.88	1.00 ± 0.001
SPCE/OH/AuNPs/MOH/GPTMS	7100 ± 86.42	277.6 ± 2.78	1.26 ± 0.0013
SPCE/OH/AuNPs/MOH/GPTMS/anti-DJ1	4520 ± 63.99	308.9 ± 2.85	2.23 ± 0.0024
SPCE/OH/AuNPs/MOH/GPTMS/anti-DJ1/BSA	5470 ± 66.07	285 ± 2.82	1.31 ± 0.0013

**Table 2:** Comparison of detection performance of the proposed biosensor with reported ELISA kits and biosensors for DJ-1 detection.

Detection Method	Linear Detection Range (ng/mL)	LOD (ng/mL)	Ref
ELISA	0.032–8	32	[33]
ELISA	0.156–10	0.094	[34]
ELISA	0.235–15	0.058	[35]
ELISA	0.781–50	0.1474	[36]
MIPPy-basedbiosensor	0.02–10	0.02	[37]
Platinum electrodes on the bio-based poly(ethylene terephthalate)	40–150	7.5	[38]
GPTMS/MOH/AuNPs@SPCEs-based biosensor	0.001–0.3	0.00059	present study

**Table 3:** Determination of DJ-1 in artificial cerebrospinal fluid samples. The table shows the spiked concentrations, detected concentrations (mean ± SD, *n* = 3), relative standard deviation (RSD%), and calculated recoveries.

Samples	Spiked Concentration (ng/mL)	Detection Concentration (ng/mL, *n* = 3)	RSD%	Recovery
aCSF–0.03 ng/mL	0.03	0.037 ± 0.0026	7.15	123.33
aCSF–0.002 ng/mL	0.002	0.0025 ± 0.0001	4	125
aCSF–0.075 ng/mL	0.075	0.074 ± 0.005	6.74	98.66
aCSF–0.25 ng/mL	0.25	0.264 ± 0.013	4.87	105.6

## Data Availability

The original contributions presented in this study are included in the article/Supplementary Materials. Further inquiries can be directed to the corresponding author.

## Supplementary Materials

The following supporting information can be downloaded at: https://www.mdpi.com/article/10.3390/bios16030146/s1, Figure S1: Optimization results of MOH concentration effects. Grey: 1%, blue: 10%, red: %5 MOH; Figure S2: Optimization results of GPTMS concentration; Figure S3: Optimization results of anti-DJ1 concentration.
