# Supplementary material for: Sensitive Detection of DJ-1 in Artificial Cerebrospinal Fluid Using a Portable GPTMS-Coordinated Gold Nanoparticle-Based Biosensor
Source: Biosensors (Basel). 2026 Mar 3;16(3):146. doi: 10.3390/bios16030146 (PMC13023608; doi:10.3390/bios16030146)
Supplement: Supplementary file 1 [file biosensors-16-00146-s001.zip › biosensors-4116876-supplementary.pdf]

biosensors

Supplementary Materials

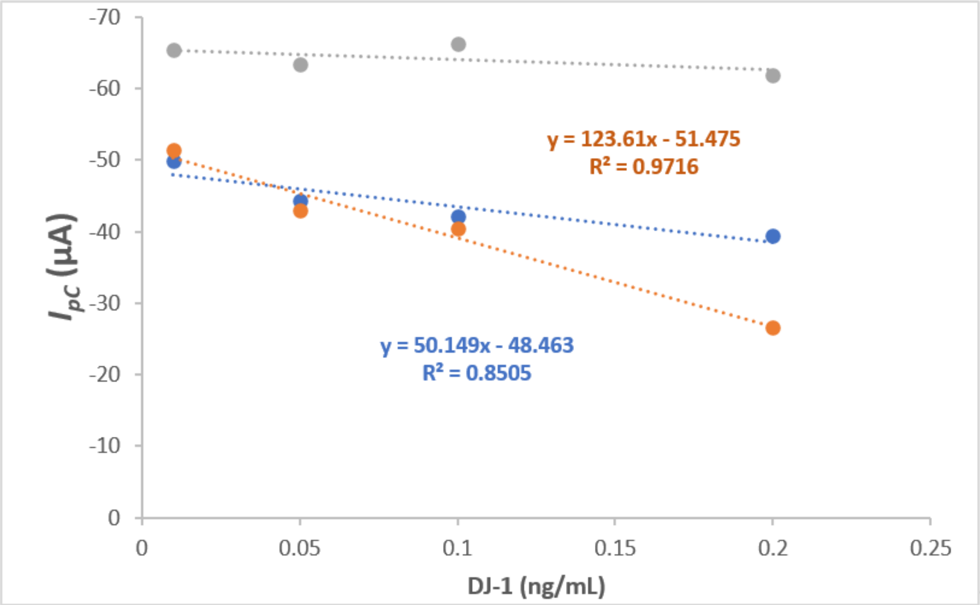

Figure S1. Optimization results of MOH concentration effects. Grey: 1%, blue: 10%, red: %5 MOH.

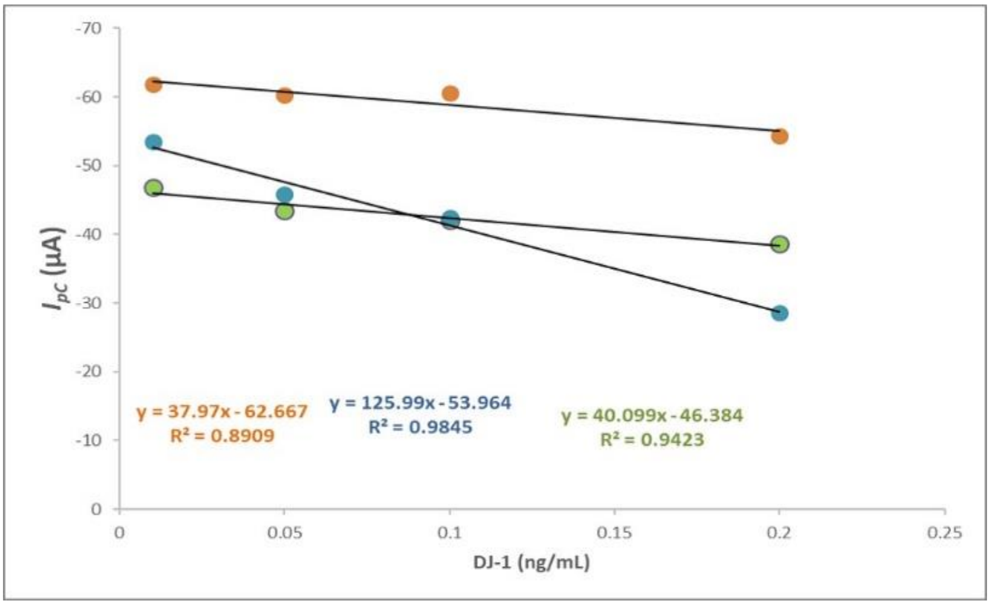

Figure S2. Optimization results of GPTMS concentration.

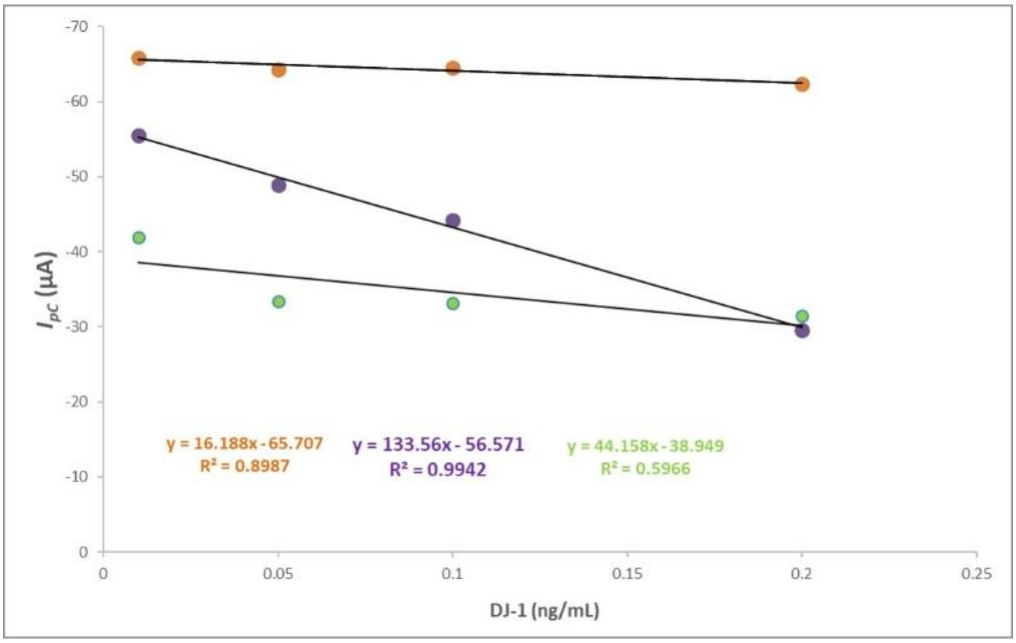

Figure S3. Optimization results of anti-DJ1 concentration.
